# Novel strategies against drug-resistant tuberculosis: a comprehensive review from molecular mechanisms to clinical translation

**DOI:** 10.3389/fimmu.2026.1912284

**Published:** 2026-10-21

**Authors:** Mingxiao Yao, Bo Pang, Ling Tian, Shuo Wang, Pengxiang Huang, Bingqin Dai, Zengqiang Kou, Shengyang Zhang

**Affiliations:** 1 Institute for Tuberculosis Prevention and Control, Shandong Center for Disease Control and Prevention, Jinan, Shandong, China; 2 Shandong Provincial Key Laboratory of Intelligent Monitoring, Early Warning, Prevention and Control for Infectious Diseases, Jinan, Shandong, China

**Keywords:** BPaLM regimen, clinical translation, drug-resistant tuberculosis, host-directed therapy, M72/AS01E, multidrug-resistant tuberculosis, myeloid-derived suppressor cells, regulatory T cells

## Abstract

In 2023, an estimated 13% of worldwide deaths resulting from antimicrobial resistance (AMR) were due to drug-resistant TB (DR-TB). The World Health Organization (WHO) documented a dramatic, sixfold increase in the number of patients treated with 6-month all-oral (BPaLM) regimens, from 5,653 patients in 2023, to 34,000 patients by 2024, while also documenting an emergence of bedaquiline resistance in real-world cohorts and a lack of efficacy of BCG revaccination in the BCG-PRIME Phase 2b trial against sustained *Mycobacterium tuberculosis* infection. Concurrently, the M72/AS01E subunit vaccine trial completed recruitment (approximately 20,000 individuals) across five high-burden countries, 11 months ahead of scheduled completion date. The first randomized controlled trial assessing DRTB-HDT host-directed therapy for DR-TB in adults (metformin + dovramilast), conducted at five international sites, is entering the late-stage readout phase. This synthesis of data, covering the period from 2015 through 2026, across six different dimensions includes molecular mechanisms of resistance, novel antimicrobial treatment modalities, host-directed therapy, immunity associated with regulatory compliance, adjuvants to vaccines, and the clinical translation of vaccine candidates. The Molecular-to-Translational continuum is used as a framework for the synthesis, with the regulatory immune function domain (T regulatory cells, myeloid-derived suppressor cells, and check-point axes) linking all five domains. Our central hypothesis is that drug resistance evolution of the microbe and failure of the host to maintain regulatory immunity are interdependent and create a bidirectional relationship with each other. We have identified nine major research gaps, with milestone dates from 2026 to 2030, based on unresolved controversies regarding the bedaquiline resistance catalog and the acquisition versus transmission narrative.

## 1 Introduction

Tuberculosis (TB) is the leading infectious cause of death worldwide (1–3). According to the World Health Organization, there will be 10.8 million new cases of TB and 1.25 million deaths caused by TB by the end of 2023; of these deaths, about 400,000 are estimated to result from drug-resistant forms of TB (AMR). About 13% of total deaths associated with AMR are attributable to TB alone. Multidrug-resistant TB (MDR-TB) is concentrated in five countries: India accounts for 27% of the global burden, while the Russian Federation, Indonesia, China, and the Philippines accounts for 7.4%, 7.4%, 7.3%, and 7.2%, respectively. Together, these five countries represent approximately 56% of the global burden of MDR-TB, but only approximately 44% of those diagnosed with MDR-TB will begin treatment in 2023. Regimen-defining events are happening in rapid succession from 2024 to 2026 (**Figure 1**). All-oral six-month regimens will be used to treat MDR-TB (including bedaquiline, pretomanid, linezolid, and moxifloxacin) with an 89% treatment success rate as compared to a 52% rate for the historical comparator standard of care (BPaLM) (4–9). Linezolid will be trialed at a final dose of 600 mg for 26 weeks (ZeNix). The number of patients treated with the all-oral six-month regimen has progressively increased from 5,653 to 34,000 as a result of the WHO update in the 2024 consolidated guideline. Success rates of 91.2% for patients treated with the all-oral six-month regimen in Nigeria are expected to be consistent with those from trials in seven countries, including endTB and endTB-Q, which reported approximately 85-91% success rates with the all-oral six-month regimen as compared to 50-65% success rates with conventional injectable regimens. However, real-world cohorts from Cape Town, South Africa, Mozambique, and Eastern Europe have indicated the emergence of acquired resistance to bedaquiline.

**Figure 1:**
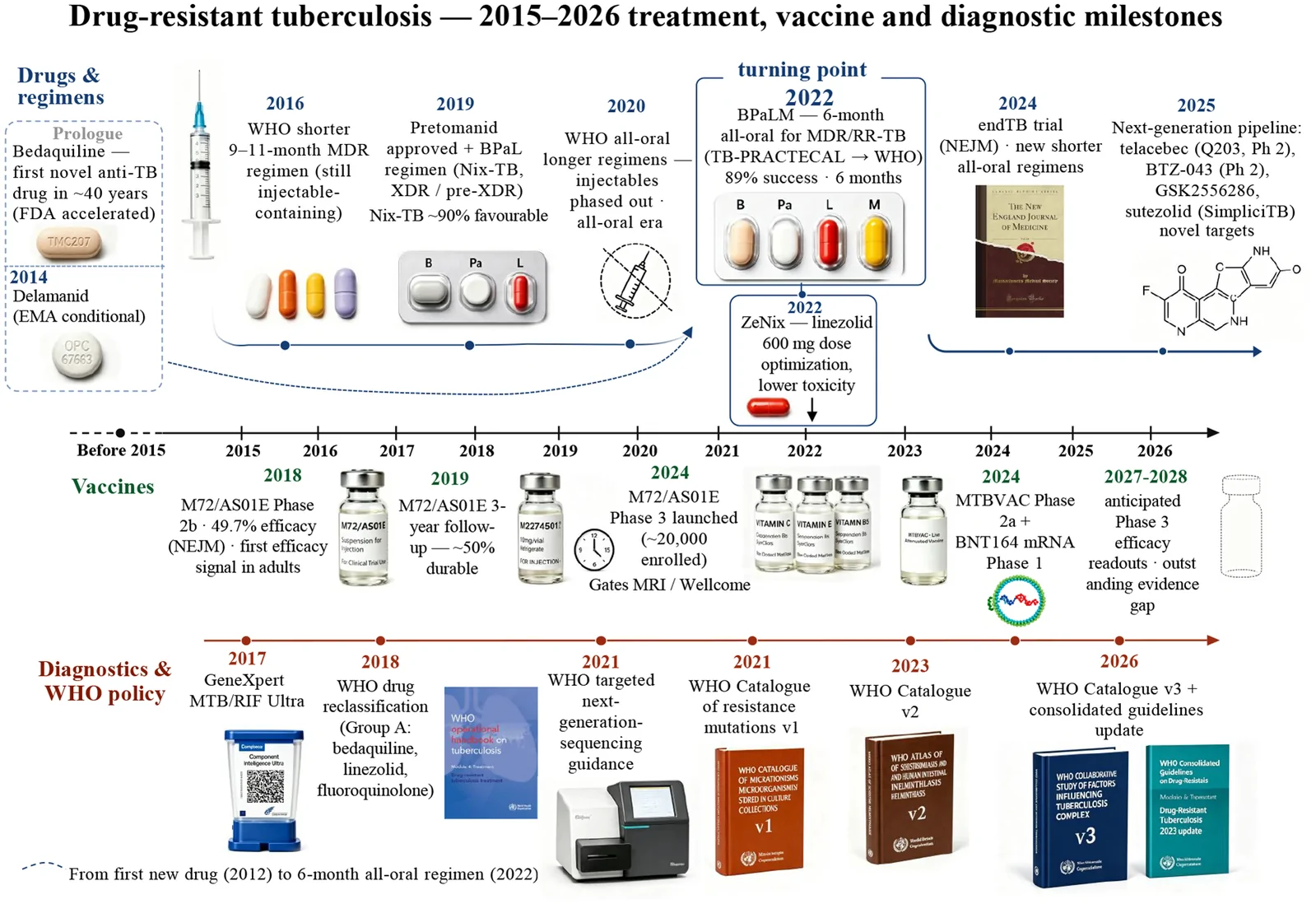
Selected DR-TB treatment, vaccine, and diagnostic milestones, 2015–2026.

Based on confirmatory trials evaluating the efficacy of BCG-PRIME and Phase 2b, it was determined that the vaccine (efficacy, -3.8%) did not offer protection against *M. tuberculosis*, with a 95% CI of -37 to 21 for IGRA-negative adolescents from South Africa, effectively eliminating the opportunity to utilize BCG as an overall vaccination strategy in this population (10). The M72/AS01E sub-unit vaccine has completed recruitment considerably in advance of schedule relative to investigators’ expectations for enrolling N 20 K adolescents/adults from Indonesia, Kenya, Malawi, South Africa, and Zambia, with the anticipated primary efficacy readout expected to occur during 2027-2028, at which time Phase 3 will have completed the full development process. However, MTBVAC will have been evaluated in Phase 3 and given to newborns in South Africa, Madagascar, and Senegal, in addition to adolescents/adults from Phase 2b and IMAGINE, respectively, and BioNTech will have initiated Phase 1 to evaluate BNT164a1/b1 mRNA TB vaccine constructs, while at the same time performing a double-blind, randomized study evaluating metformin plus dovramilast in South Africa, Kenya, Zambia, Malawi, and Senegal, which will represent the first study of an HDT-based approach [i.e (11), host-directed therapy] to provide evidence for the late-stage efficacy of a proposed DR-TB-specific HDT (12).

There is an emerging trend to review all evidence for an infection according to the management pillar, resistance mechanisms, vaccine candidates, and type of compound. Our review uses a six-branch continuum as an organizing framework and proposes, rather than establishes, a bidirectional-reinforcement hypothesis linking bacterial resistance evolution with host regulatory immunity. The coexistence of persister reservoirs, Treg/MDSC enrichment, and chronic antigenic load is consistent with this hypothesis but does not demonstrate causal feedback or establish the benefit of combined interventions. The remainder of this document discusses the remaining six branches and any areas where there is disagreement in the evidence that has been developed from 2024–2026 and concludes with information on the nine unanswered questions related to named milestones through 2026 and into 2030 (3, 13–15).

## 2 Background: the drug-resistant tuberculosis burden

### 2.1 Epidemiology and terminology

Between 2015 and 2023, the proportion of newly diagnosed patients with rifampicin- or multidrug-resistant TB decreased from 4.7% to 3.2%. The total number of individuals with TB remains significant; however, 18% of previously treated patients have MDR-TB occurrences compared to 3.3% of new diagnoses. We use WHO terminology throughout: rifampicin-resistant TB (RR-TB); multidrug-resistant TB (MDR-TB), resistant to rifampicin and isoniazid; pre-extensively drug-resistant TB (pre-XDR-TB), MDR/RR-TB with resistance to any fluoroquinolone; and extensively drug-resistant TB (XDR-TB), pre-XDR-TB with additional resistance to bedaquiline or linezolid (16, 17). In all uncomplicated rates of success for the treatments currently being used for MDR-TB, the pooled success rate is approximately 85.0% (1, 2, 16). In pre-XDR-TB treatments, the pooled success rate is approximately 75.0%. In complicated XDR-TB treatments, the pooled success rate is approximately 65.0%.

### 2.2 Resistance acquisition versus transmission

Recent genomic evidence suggests that a major cause of DR-TB in regions with high rates of disease is transmission and that there have been long-standing debates about whether initial acquisition of *de novo* or continuous spread of pre-existing resistant strains of the pathogen has led to epidemics caused by the emergence of DR-TB. However, resistance may also be acquired from inadequate or subtherapeutic treatment. Mutations in the *rpoA*, *rpoB*, and *rpoC* RNA polymerase subunits of rifampicin resistant isolates will allow these isolates to circulate at rates comparable to those of wild-type or normal isolates (18, 19). The Eurasian Lineage 4.2 Ural demonstrates that compensated MDR-TB transmission has occurred through Moldova, Ukraine, and Belarus, where MDR infections were approximately 24 to 54% of new TB cases (20, 21). A genome-wide association analysis by lineages of DR-TB demonstrated that 2.8 to 4.7% of DR-TB isolates had mutator phenotypes, indicating that the presence of these phenotypes led to greater than a four-fold higher rate of acquiring resistance as a result of exposure to less than optimally effective therapy (i.e., subtherapeutic) for the *de novo* pathogen. This has significant implications for controlling DR-TB in areas of high disease burden; thus, the optimum way to control DR-TB in areas where there are high numbers of DR-TB-infected individuals is to stop the transmission of DR-TB, and/or in combination with the use of pharmacologic therapy, will require the development of immunomodulatory interventions and vaccine-based interventions through the continued development of DR-TB (22).

## 3 Synthesis framework and methodology

This evidence base for the years 2015–2026 is categorized in accordance with a continuum running from Molecular to Translational, with Branch 4 (Regulatory Immunity) linking the other five branches (**Table 1**; **Figure 2**) of the continuum. Branch 1 (MMR; [WHO Catalogue]) consists of mechanisms of resistance including molecular changes (e.g. mutation and compensatory evolution), efflux pumps, adaptive responses (within-host micro-evolution), and lineage-based pathways. Branch 2 consists of new classes of antimicrobial therapy sorted by energy metabolism, ribosome (DprE1) and cell-wall chemistry, nitroimidazoles (F420), and cholesterol catabolism (AMPK). Branch 3 reorganizes HDT based on host pathways rather than compounds: e.g. autophagy (AMPK) and cytokines and checkpoints (Treg & MDSC, respectively). Branch 4 develops regulatory and immunomodulatory scaffolding (DR-TB) as well as the regulatory pathway (BCG-PRIME) for the product. Branch 5 contains the pipeline of vaccine products supported by evidence from the perspectives of Regulatory Immunity (M72/AS01E; MTBVAC; BNT164 for mRNA vaccine) with respect to adjuvants. Branch 6 supports the translation into clinical practice of the multi-center cohort evidence from the following studies: TB Portals (33,000+ patients in 16 countries), CRyPTIC (15,211 isolates from 23 countries), ReSeqTB, endTB, TB-PRACTECAL, STREAM, and IeDEA (23, 24). Each section of the evidence base outside of Branch 4 contains a minimum of two explicit and accurate cross-references to Branch 4 (one relates to mechanisms and one relates to therapies) to provide a vertical spine architecture between all sections of the evidence base and Branch 4. 

**Table 1:** Six-branch taxonomy summary: molecular-to-translational continuum with regulatory immunity as the vertical spine.

Branch	Layer	Definition	Key targets/pathways	Representative agents	N papers	Translational stage
1	L1 horizontal	Molecular resistance mechanisms	*rpoB*/*katG*/*gyrA* mutations; efflux pumps; compensatory evolution	N/A (mechanism layer)	81	Foundational
2	L2 horizontal	Novel antimicrobial therapeutics	ATP synthase; cytochrome *bc1*; DprE1; ribosome; F420	BPaL, BPaLM, BDLLfxC, telacebec, BTZ-043, GSK2556286	101	Phase 1–3 + approved
3	L3 horizontal	Host-directed therapy	Autophagy, AMPK, MMP, cytokines, checkpoint	Metformin, dovramilast, statins, vitamin D	56	Phase 2–3 (DRTB-HDT)
4	L4 **vertical spine**	**Regulatory immunity & vaccine adjuvants**	**Treg/MDSC/check point axes**	**AS01E, GLA-SE, anti-CD25, ATRA, TLR agonists**	**57**	**Preclinical–Phase 1**
5	L5 horizontal	Vaccine pipeline	Subunit/live-attenuated/mRNA + adjuvants	M72/AS01E, MTBVAC, BNT164, H56:IC31, ID93+GLA-SE	52	Phase 1–3
6	L6 horizontal	Clinical translation	Real-world implementation; equity; subgroup MCID	WHO BPaLM scale-up, BDLLfxC alternative, prevention regimens	144	Implementation

Bold values highlight Branch 4, the vertical spine of the framework.

**Figure 2:**
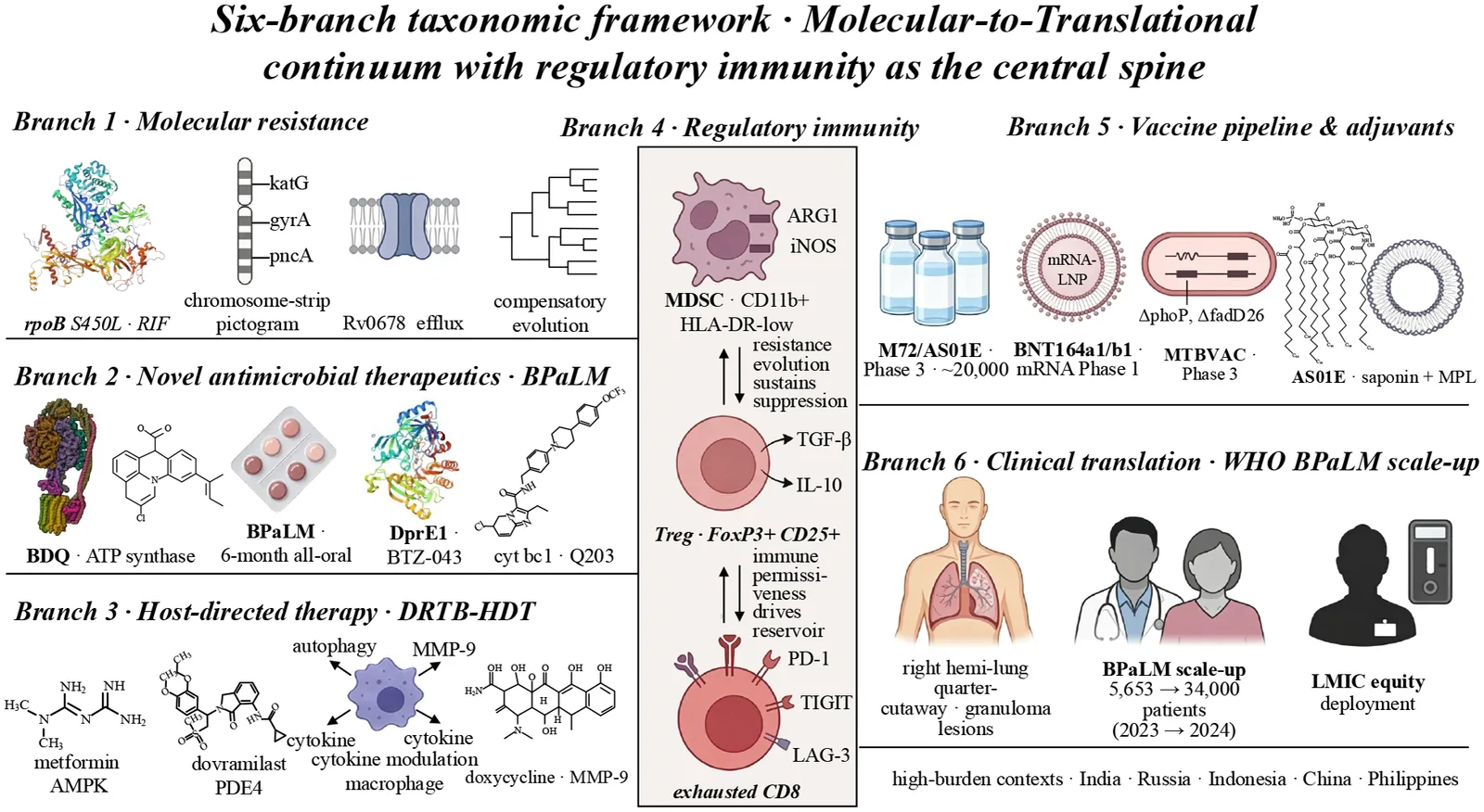
Six-branch molecular-to-translational framework with regulatory immunity as the central spine.

The six-branch structure is an interpretive framework rather than a demonstrated causal architecture; links to Branch 4 are therefore treated as evidence-supported, indirect, or hypothesis-generating according to the underlying studies. According to the conventions outlined in PRISMA-ScR, this synthesis was created using a methodology described as a “narrative systematic hybrid”. The following databases were searched: PubMed/MEDLINE; Embase; Web of Science; Scopus; Cochrane Central Registry; and linkages to records of clinical trials (ClinicalTrials.gov, WHO International Clinical Trials Registration Platform, TB Portals, CRyPTIC, and ReSeqTB), as well as authors’ servers for preprints (bioRxiv and medRxiv). In the follow-up analysis of the six high-impact reviews, 858 citations were obtained. The citations’ duplicate results (using DOI and title/abstract screens) identified 612 records; 414 underwent full-text assessment and 350 were eligible for qualitative synthesis for the period 2024–2026 (**Figure 3**; see **Supplementary Table SA-2**). Cochrane Risk of Bias 2 was used for randomized studies, ROBINS-I was used for observational studies, and AMSTAR-2 was used with regard to reviews conducted previously. SYRCLE and GRADE provide identification of branch level of narrative certainty. This synthesis only includes evidence from peer-reviewed literature or preprint quality-assured databases, with no collection of raw patient level data. Searches covered 1 January 2015 to 30 June 2026; database-specific terms combined tuberculosis, drug resistance, treatment, host-directed therapy, immunity, vaccines, and translation. Duplicate removal used DOI followed by title/abstract comparison; independent dual screening and disagreement resolution were not prospectively documented, and no protocol was registered. Here, “narrative systematic hybrid” denotes systematic searching followed by narrative synthesis; preprints were appraised separately and were not used alone for definitive clinical conclusions.

**Figure 3:**
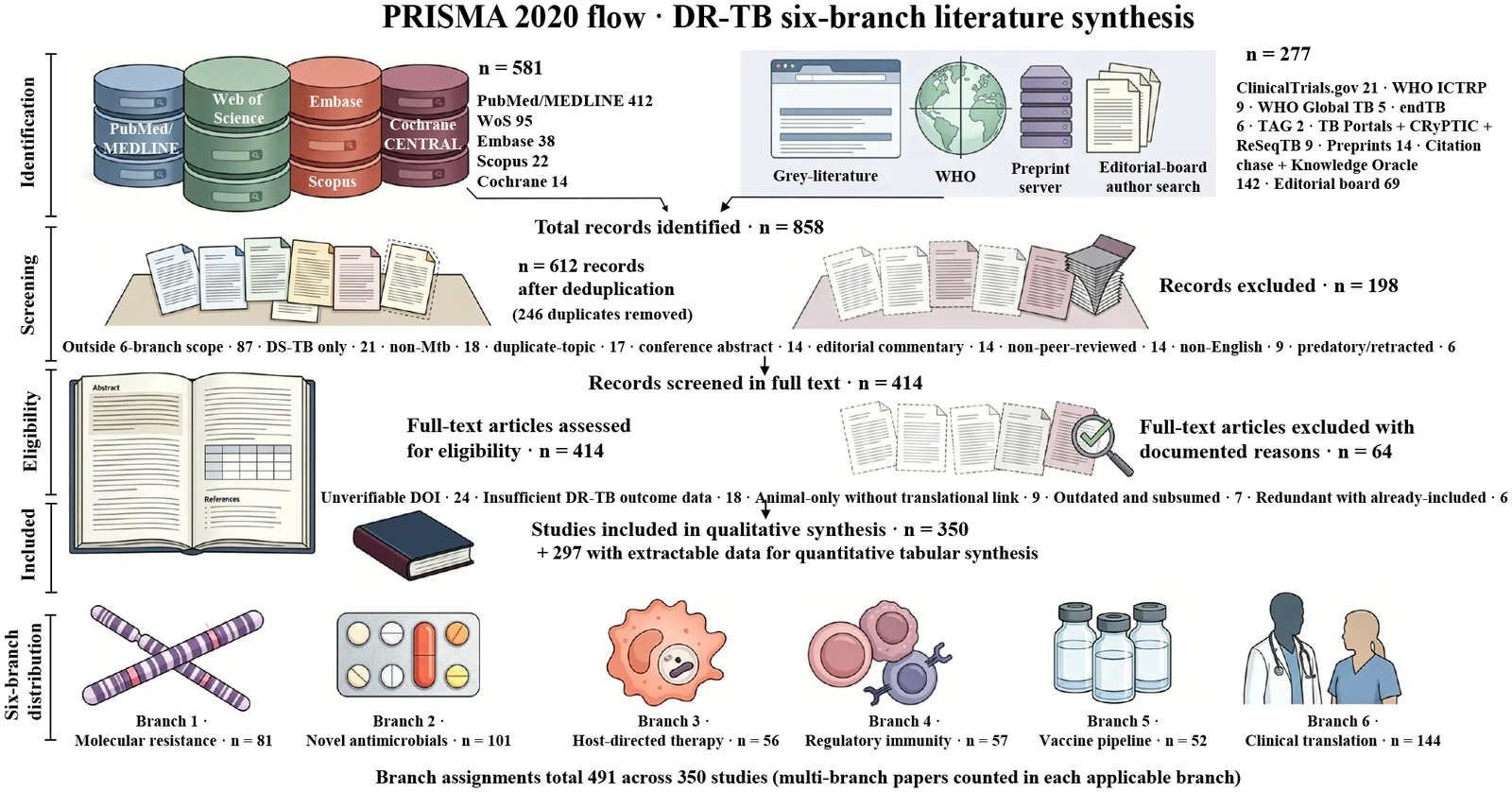
PRISMA 2020 flow diagram for the six-branch literature synthesis.

## 4 Branch 1: molecular resistance mechanisms

### 4.1 Target-gene mutation catalog (WHO v2)

The Catalogue of Mutations (2nd edition), published in 2023, provides comprehensive whole-genome sequencing predictions of drug resistance to 13 different drugs (23, 25). The first-line (rifampicin, isoniazid, ethambutol, and pyrazinamide) catalog demonstrates FIND5% concordance between the genotype and phenotype for the drugs meeting targets set forth in WHO/10. There are still numerous unresolved mutations associated with the new drugs (bedaquiline,021,022,023), as 35%-45% of the clinical variants (*atpE*, *Rv0678*, *pepQ*) have an uncertain classification; the 2nd ed (26, 27) of the CRyPTIC MICS database provides the basis for the 3rd ed. (anticipated 2027-2028) with 15,211 isolates. Clinical sputum nanopore sequencing using a clinical timescale is now available and may further assist in closing the diagnostic stewardship gap for second-line agents in under-resourced areas (27–30).

### 4.2 Compensatory mutations and transmission-driven resistance

Compensatory mutations have made it possible for rifampicin-resistant lineages to restore their growth rates and thereby expand through human contact and circulate at the same rate as wild-types. The broad distribution of a virulence-linked haplotype among 17,641 isolates supports an important role for transmission but does not exclude within-host acquisition of resistance. The expansion of the Ural sublineage 4.2 in Eastern Europe and the transcontinental expansion of the Beijing lineage 2 exemplify these evolutionary processes. These findings support transmission control; any additional benefit from vaccine or host-directed interventions remains to be established (20, 22, 31, 32).

### 4.3 Efflux pumps, mutator phenotypes, and within-host evolution

Overproduction of efflux pumps may result in a “pre-resistance” state that does not have heritable properties and cannot be identified by traditional minimum inhibitory concentration tests. The efflux pump MmpL5 is regulated by *Rv0678*, thus resulting in resistance to bedaquiline and clofazimine. Meanwhile, *Rv1258c* (Tap) has been associated with isoniazid and streptomycin tolerance (4, 33). Mutator phenotypes associated with mutations in DNA mismatch repair, nucleotide excision repair, and oxidative damage repair pathways are present in 3.4% of Lineage 4, 2.8% of Lineage 2, and 4.7% of Lineages 5 and 6 strains (34), all of which are enriched in patients with treatment failure (22, 35). Polyclonal diversification caused by subtherapeutic dosing generates tolerant subpopulations that later acquire heritable resistance due to compensated fitness pathways. There are multiple separate selective pressures to select for resistant organisms from three separate physical compartments of the granuloma (i.e., the core of the granuloma is hypoxic; the caseum is necrotic; and the cellular rim) (36, 37).

### 4.4 Lineage-specific resistance trajectories and the bidirectional cross-link

Lineage 2 strains of Beijing have both the compensated rifampicin-resistant haplotype and the very high baseline mutation rates. They are therefore over-represented in terms of their contribution to the amounts of MDR-TB found in China, the Russian Federation, and Eastern Europe (15, 32). Lineage 4.2 of Ural is currently expanding into 24 countries (21). The results from the study of 131,753 strains from Africa show a large degree of overlap in the assignment of *rpoB*-resistance haplotypes as well; in addition, there is less within-lineage clustering of haplotypes compared with Eurasian lineages (38, 39). For the Latin American Lineage 4 subclades, the evolutionary pathways differ from those of the Eurasian lineages. Differences in the geographic distribution of bedaquiline resistance mutations are shown in that the *Rv0678* and *pepQ* haplotypes dominate in Cape Town; the *atpE* haplotypes dominate in the Russian Federation and Eastern Europe; and independent acquisitions have been made from multiple genetic backgrounds in Mozambique. Therefore, it will be important to develop diagnostic stewardship strategies for the ongoing evolution of resistance based on regional differences rather than using a single universal mutation panel (5, 40).

Granuloma single-cell data associate hypoxia and MDSC–Treg enrichment with restricted effector-cell access and persistent antigen load. Whether these features interact reciprocally or justify combined antimicrobial and immune-directed treatment remains uncertain (41, 42).

## 5 Branch 2: novel antimicrobial therapeutics

### 5.1 Approved agents and the BPaL/BPaLM revolution

Four main transitions within TB therapy are expected to dramatically alter the way TB is treated from 2024 onwards, i.e., (1) moving WHO to BPaLM and BPaL into first-line, all-oral, 6-month treatment regimens; (2) establishing energy metabolism inhibition as a distinct category of mechanisms; (3) developing DprE1/GSK2556286 and entering Phase 1b to 2b studies with next-generation oxazolidinones as backup treatment options; and (4) identifying bedaquiline-resistant cohorts in patients being treated with bedaquiline (7, 43–45). Bedaquiline, a Diarylquinoline, is known to inhibit the mycobacterial ATP Synthase via its binding to the sink face of the ATP Synthase crystal structure–identified by cryo-Electron microscopy of the sole-energy-producing mycobacterial ATP Synthase, whereby BPaL/BPaLM has a sterilizing effect against non-growth replicating persisters (**Figure 4**). In addition, pretomanid (a nitroimidazole) activates via F420 reductive, leading to the production of reactive nitrogen species against Mtb; because of the differing mutation patterns of *ddn*, *fbiA-D*, and *fgd1*, there are four classes of TB-resistant isolates not found in current standard drug susceptibility testing panels (29, 46). Linezolid (oxazolidinone) has been finalized in ZeNix as daily doses of 600mg for 26 weeks (grade Mtb peripheral neuropathy was identified in a meta-analysis of 2269 patients at an incidence of 1.8%). Further, delamanid and clofazimine complete the Group A and Group B inventories and have the potential for cross-resistance via the actions of the *Rv0678*/MmpL5 efflux pathway with respect to bedaquiline (33, 47).

**Figure 4:**
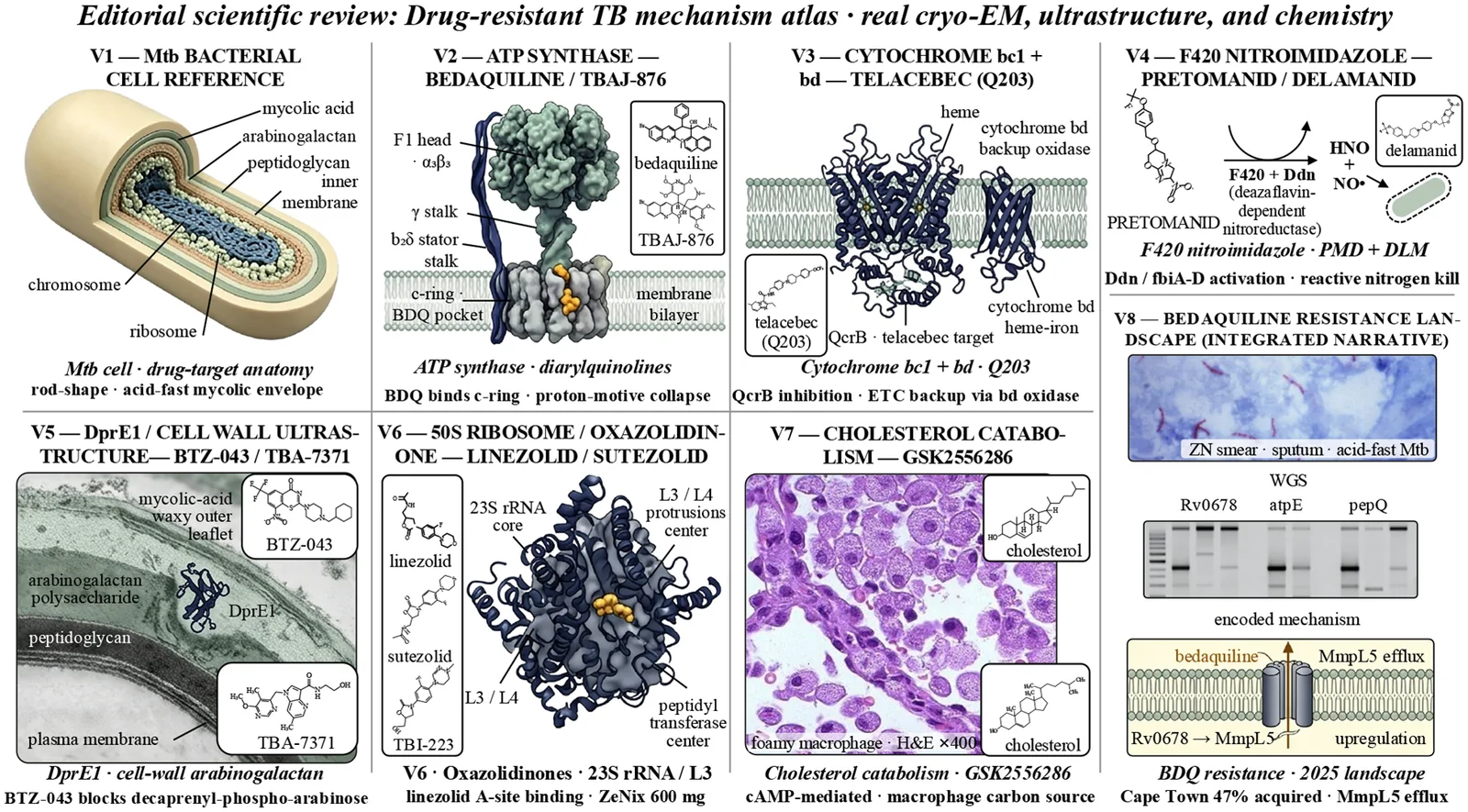
Drug-target mechanism atlas for drug-resistant tuberculosis.

A total of 552 patients were randomly assigned to this study (TB-PRACTECAL) from four countries (Belarus, South Africa, Uzbekistan, and Pakistan) to receive either the intervention (BPaLM) or the control (WHO). There was a statistically significant difference in the proportion of patients who succeeded with the study drug (89%) compared to the control drug (52%) (6). The endTB multi-country study demonstrated comparable treatment success rates for all five all-oral regimens when used for 9 months, including among patients with culture conversion prior to receiving treatment with the study regimen or the non-study regimen. The Nigerian National TB Implementation Cohort demonstrated treatment success of 91.2%. The 38-cohort meta-analysis demonstrated treatment success rates for the control group (BPaLM) of 85%-91%, with a significant reduction in mortality (16.4%-7.5%) for patients who received injected versus control treatment (**Table 2**) (48). Both the economic evaluations for TB-PRACTECAL and modeling in Moldova demonstrate cost-effectiveness and/or cost savings for BPaLM; however, there are limitations imposed upon the drug cost-based treatment model for BPaLM due to insufficiencies in diagnostic capacity and programmatic support (49, 50). The remaining treatment failures are disproportionately represented by patients who were previously diagnosed with extensive lung disease, have a HIV co-infection, are undernourished, and/or who meet the Treg/12 immunophenotype that provides justification for use of adjunctive HDT in Branch 3 (8, 9, 42, 51).

**Table 2:** Drug-resistant tuberculosis treatment regimens: indications, duration, and outcomes from pivotal trials and real-world cohorts.

Regimen	Indication	Dur. (mo)	Trial	Year	n	Success (%)	Mort. (%)	Key AE	Reference
BPaL	MDR/pre-XDR/XDR (FQ-S)	6	ZeNix (LZD 600 mg)	2022	181	89.0	6.6	Neuropathy; myelosuppression	(7)
BPaLM	MDR/RR	6	TB-PRACTECAL	2022	552	89.0	1.8	Hepatotoxicity; neuropathy	(6)
BPaLM	MDR/RR (real-world)	6	Nigeria pilot	2025	460	91.2	4.0	As trial profile	(48)
BPaLM	MDR/RR (India NTEP)	6	Uttar Pradesh + multi-state	2024	121,000	78.0 (6-mo)	5.2	As trial profile	(92)
BDLLfxC	MDR/RR pregnancy/FQ-S	9	endTB	2024	754	84.0	3.1	Hepatotoxicity; QTc	(8)
BDLLfxC	pre-XDR FQ-R	6–9	endTB-Q	2025	324	75.0	4.0	As BDLLfxC	(9)
9-mo all-oral	MDR/RR	9	STREAM Stage 2	2022	588	83.0	7.0	As BDLLfxC	(90)
Legacy long (injectable)	MDR/RR (deprecated)	18–24	Pre-2019 cohorts	2015–19	282	50–65	16.4	Hearing loss; renal	(91)
LFX prevention	MDR contacts	6	VQUIN MDR	2024	21,041	87 (no progression)	0.4	GI; tendon	(94)
BPaLM (pediatric<14 y)	MDR/RR pediatric	6–9	ATS pediatric brief	2024	168	88.0	2.8	As trial profile	(92)

### 5.2 Pipeline diversification: energy metabolism, DprE1, and cholesterol catabolism

Telacebec (also known as Q203) is an imidazopyridine-class cytochrome bc1 inhibitor that has completed clinical evaluation in pulmonary TB, showing early bactericidal activity. This confirmed clinical proof-of-concept that cytochrome bc1 is a viable therapeutic target and established a rationale for developing dual cytochrome bc1\bd combinations to eliminate antibiotic-tolerant M. tuberculosis (52). BTZ043, a DprE1 inhibitor (benzothiazinone), has completed Phase 1b/2a clinical trials in South Africa, for pulmonary TB with proven clinical bactericidal activity and pharmacokinetics supporting the lesion level; thus Phase 2b is ready for further clinical studies. GSK2556286 is mediated by cAMP, which targets cholesterol breakdown by an entirely different mechanism than ATP synthase and DprE1, and has been shown to significantly reduce the amount of time required for the use of combination therapies with bedaquiline and pretomanid in murine models (53–57). Next-generation oxazolidinones, such as sutezolid in SUDOCU-01, delpazolid in DECODE-01, and TBI-223, provide linezolid as backup compounds with a therapeutic window. Also, next-generation diarylquinolines such as TBAJ-876 and TBAJ-587 have activity against *Rv0678* mutant strains and demonstrate better QT interval prolongation than existing/older therapies. Repurposing platforms based on *Mtb* genome-scale metabolic modeling and AlphaFold target validation will provide a pipeline for the rapid development of drugs/therapies that effectively combat the development of drug resistance against bedaquiline (58, 59) (**Table 3**).

**Table 3:** Novel anti-tuberculosis therapeutics: targets, mechanisms, and drug-resistant tuberculosis activity (2012–2026).

Drug	Class	Primary target	Phase	Resistance mechanism	Efficacy/endpoint	Role
Bedaquiline	Diarylquinoline	ATP synthase (*atpE*)	Approved 2012	*Rv0678*, *atpE*, *pepQ*	BPaL/BPaLM backbone	Backbone
Pretomanid	Nitroimidazole	F420/Ddn/DprE1 pathway	Approved 2019	*ddn*, *fgd1*, *fbiA-D*	ZeNix 6-mo 89%	Backbone
Linezolid	Oxazolidinone	23S rRNA/50S ribosome	Approved	*rrl*, *rplC*	600 mg × 26 wk	Backbone
Delamanid	Nitroimidazole	DprE1/F420	Approved 2014	*ddn*, *fbiA-D*	MDR cure 85%	Alternative
Clofazimine	Riminophenazine	Outer membrane/NADH oxidase	Approved	MmpL5 efflux (*Rv0678*)	STREAM efficacy	Backbone (BDLLfxC)
Moxifloxacin	Fluoroquinolone	DNA gyrase	Approved	*gyrA*, *gyrB*	TB-PRACTECAL	Backbone
Telacebec (Q203)	Imidazopyridine	Cytochrome *bc1* (QcrB)	Phase 2	*qcrB*	Phase 2a EBA signal	Pipeline
BTZ-043	Benzothiazinone	DprE1	Phase 1b/2a	*dprE1*	Phase 1b activity	Pipeline
GSK2556286	Cholesterol catabolism	cAMP/cholesterol	Phase 1	N/A	EBA pending	Pipeline
TBAJ-876/TBAJ-587	Next-gen diarylquinoline	ATP synthase	Phase 1	Parallel to BDQ	Murine combos active	BDQ successor
Sutezolid/Delpazolid	Next-gen oxazolidinone	23S rRNA	Phase 2	Pipeline class	Lower neuropathy than LZD	LZD successor
Metformin (HDT)	Biguanide	AMPK/autophagy	DRTB-HDT RCT	N/A (host target)	5-country RCT in progress	HDT adjunct
Dovramilast (HDT)	PDE4 inhibitor	cAMP/macrophage	DRTB-HDT RCT	N/A	5-country RCT in progress	HDT adjunct
M72/AS01E	Subunit + AS01E	Mtb32A + Mtb39A fusion	Phase 3 enrollment complete	N/A	Phase 2b 49.7% VE	Vaccine
MTBVAC	Live-attenuated (*phoP*/*fadD26* del)	Whole-cell + cell-mediated immunity	Phase 3 + IMAGINE	N/A	Infant immunogenicity > BCG	Vaccine
BNT164a1/b1	mRNA-LNP	Multi-antigen (ESAT-6, CFP-10, Ag85B)	Phase 1	N/A	Immunogenicity pending	Vaccine

### 5.3 Emerging bedaquiline resistance and the operational response

Patients in Cape Town who do not have an adequate response to bedaquiline-based therapy exhibit a prevalence of acquired bedaquiline resistance of 47%. The mutations *Rv0678* and *pepQ* together comprise this high resistance rate, but *atpE* is more prevalent in Eastern Europe and other areas, as illustrated by independent acquisition from different genetic backgrounds as documented in the Mozambique reference data (4, 5, 40) (see **Supplementary Table SA-1**). The *atpE* mutation affects bedaquiline binding to the bedaquiline receptor and confers very high levels of bedaquiline resistance. *Rv0678* and MmpL5 efflux result in lower levels of bedaquiline resistance (but only when cross-resistance to clofazimine is present). Next-generation diarylquinolines are effective against *Rv0678*-containing organisms, but the efficacy of next-generation diarylquinolines in salvaging *Rv0678* varies by region. Because of emerging prevalence rates, a forward-looking operational strategy can be established which includes the following clinical workflow for patients: (1) pre-treat patients with whole-genome sequencing (or targeted amplification sequencing) of DST (including nanopore sequencing from sputum); (2) maintain next-generation diarylquinoline contingency plans; and (3) utilize mechanism-orthogonal therapy (telacebec, GSK2556286, BTZ-043) followed by a Branch 3 hybridized-directed therapy (HDT) in order to globally reduce bacterial loads and thereby diminish selective pressure. The published rates of *ddn* and *fbiA-D* resistance to pretomanid are below threshold in most published studies comparing geographic variation, and *ddn* and *fbiA-D*remain undetectable by the standard DST assay. Energy-metabolism blockade may alter immune phenotypes, but the proposed link to Branch 4 is indirect and patient-dependent (13, 28, 60).

## 6 Branch 3: host-directed therapy

Host-directed therapy is being investigated as an adjunct to address persister biology and post-treatment immune dysfunction (**Figure 5**). Granuloma immune regulation is one proposed target, but its contribution to bacterial persistence and the value of immune-phenotype stratification remain uncertain (61–64).

**Figure 5:**
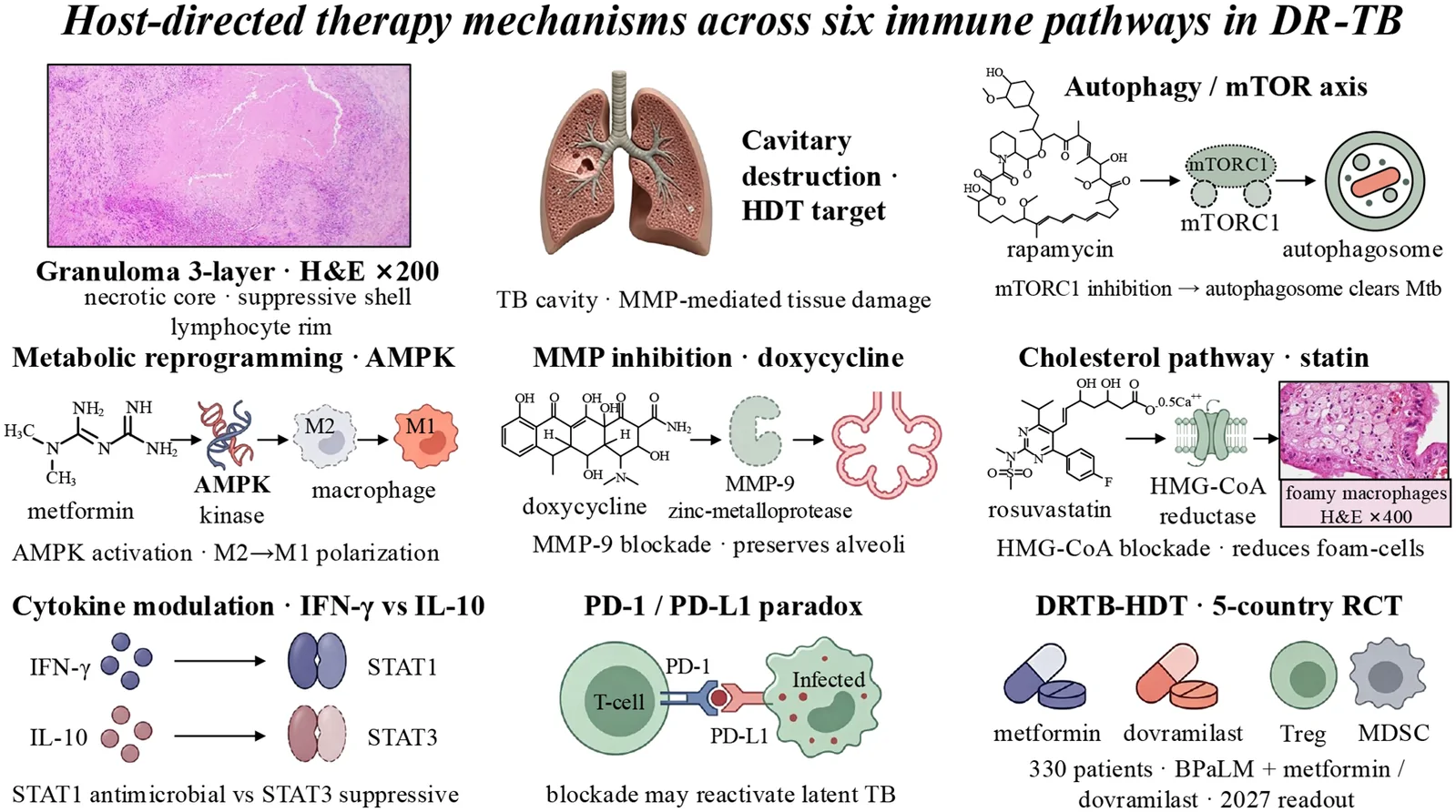
Host-directed therapy mechanisms across six immune pathways in DR-TB.

The activation of AMPK in macrophages by metformin is associated with increased killing of Mtb, enhanced M1 polarization of macrophages, and altered mitochondrial reactive oxygen species generation through the IFN-γ unconditional killer pathway (65–69). Evaluation of the efficacy of standard BPaL/BPaLM combined with metformin or the phosphodiesterase-4 inhibitor dovramilast compared to standard BPaL/BPaLM alone is ongoing in DRTB-HDT, a randomized controlled trial occurring in multiple countries (Romania, Moldova, Georgia, Mozambique, and South Africa; n≈330). The trial’s primary endpoints of forced expiratory volume in 1 second (FEV1) recovery and time (in months) to conversion of sputum culture will be evaluated at months 6 and 18 (13). The sites included three of the Ural lineages (lineage 4.2, Beijing and African lineage 4) (see **Supplementary Table SB-2**), and only nondiabetic patients will be included in the metformin treatment group to evaluate any direct impacts of Mtb independent of the possible effects of glycemic control. Research has shown that use of rapamycin or everolimus to inhibit the activation of mTOR stimulates the initiation of autophagy in murine models (70) and reduces the burden of Mtb within macrophages. Additionally, one study demonstrated that the use of adjunct rosuvastatin will reduce time to conversion of sputum culture among adults infected with rifampicin by DR-TB, thus providing a plausible mechanistic mechanism on which to base future clinical applications. Administration of vitamin D promotes earlier conversion of sputum culture for patients with levels below the lower limit of normal as measured by the LL-37 pathway of cathelicidin. The use of HDT with cytokine modulation requires caution because TNF blockade may reactivate clinically defined latent TB infection; latency should not be inferred from bacterial persistence or stable burden alone. There are other recently developed adjunctive modalities for treating Mtb, such as granzyme-A-mediated CD8, OX40 receptor agonist function in regard to CD4 dysfunction due to checkpoint (KIR)-related defects, and the use of Fc-engineered antibodies for recruiting neutrophils for Mtb killing. Preclinical studies link several HDTs to regulatory-cell pathways, but these effects are not established across all HDTs or in DR-TB patients. DRTB-HDT may test selected clinical implications of the framework but cannot by itself establish bidirectional reinforcement (71).

## 7 Branch 4: regulatory immunity and vaccine adjuvants

### 7.1 Treg, MDSC, and checkpoint axes in granuloma immunology

Single-cell and spatial-transcriptomic atlases describe a hypoxic MDSC/Foxp3-rich core, an osteopontin-expressing myeloid interface, and an outer lymphocyte layer. These spatial associations do not by themselves establish causal immune suppression (36, 37). Treg and MDSC enrichment is associated with restricted effector-cell access, but its causal role in maintaining the bacterial niche remains uncertain (14, 41, 72–74). The trans-sialidase-targeted vaccine system created in *Trypanosoma cruzi* serves as a mechanism to aid in the development of future Treg-modulating vaccines. The biology of checkpoint molecules regarding TB is bidirectional; the dysregulation of the expression of PD-1/PD-L1 results in the reactivation of patients with oncological diagnoses who are treated with immune checkpoint inhibitors, while the limited duration that checkpoint inhibitors are used may lead to the re-sensitization of exhausted CD8 T cells of patients with chronic DR-TB (61, 62). Blockade of TIGIT improves control of the Mtb infection compared to controlling PD-1 blockade by the mice.

### 7.2 The bidirectional reinforcement model and adjuvant-design framework

The component associations underlying the bidirectional-reinforcement hypothesis are evidence-supported, whereas the proposed cross-branch feedback remains an indirect, hypothesis-generating interpretation (42). Chemical control alone may not address regulatory-immune suppression, but the need for combined antimicrobial, immune-directed, and vaccine strategies requires direct testing.

Regulatory-cell-aware vaccine design may be informed by adjuvant chemistry, although this strategy remains investigational. For example, AS01E promotes Th1-biased responses through its liposomal saponin and monophosphoryl lipid A components; however, the M72/AS01E Phase 2befficacy estimate does not establish Treg modulation as the mechanism of protection (75). Saponin-only formulations like TQL1055 may identify saponin as a component of an adjuvant in preclinical studies and Mtb challenges. Additionally, STING, manganese-based sensitizers (IC31), and cationic peptides (TLR9) are regulatory-cell-aware adjuvants that are currently undergoing preclinical and early clinical evaluation, all of which interact with cGAS (76). It is possible that lipid nanoparticles may provide some adjuvanticity because mRNA-based (LNP) vaccines use them to create the design logic for BNT164a1/b1 (77). Co-administering checkpoint inhibitors (anti-CTLA-4 and transient Treg suppression) and other regulatory immune modulators for the DR-TB-specific pipeline (ATRA-mediated MDSC differentiation, low dosing of 5-fluorouracil MDSC depleting, tadalafil modulating PDE5) is outlined in **Supplementary Table SB-3**. Phenotype-stratified basket trials could test these proposed combinations in DR-TB, but no such regulatory-cell-modulator trial has yet established clinical feasibility or efficacy (Section 11) (78).

## 8 Branch 5: vaccine pipeline

The late-stage TB vaccine readouts occurring between 2024 and 2026 represent the highest density of late-stage TB vaccine readouts occurring after BCG was released (**Table 4**). The pipeline is organized based on regulatory-immuno-adjuvant design with a view toward complementing the reviewer-organized reviews of the *npj Vaccines* program (79).

**Table 4:** Drug-resistant tuberculosis milestone timeline, 2015–2026.

Year	Type	Event	Key reference	Impact
2018	Vaccine	M72/AS01E Phase 2b reports 49.7% efficacy	(75, 83)	First subunit signal >50%
2019	Drug	Pretomanid FDA approval as part of BPaL regimen	(29)	XDR-TB regulatory anchor
2021	Policy	WHO updated pre-XDR/XDR definitions	(17)	Resistance restratification
2022	Trial	TB-PRACTECAL BPaLM 89% vs SOC 52%	(6)	BPaLM regulatory case
2022	Trial	ZeNix linezolid 600 mg × 26 wk preferred	(7)	LZD dose optimization
2023	Cohort	Cape Town 47% acquired bedaquiline resistance	(4)	BDQ durability concern
2023	Surveillance	WHO Catalogue of Mutations v2 (13 drugs)	(23)	Molecular DST anchor
2024	Policy	WHO 2024 guideline: BPaLM first-line MDR/RR-TB	(85)	6-month standard of care
2024	Trial	endTB Phase 3 across seven countries	(8)	9-month alternative
2024	Implementation	BPaLM scale-up 5,653 → 34,000 patients (6×)	(88)	Largest single-year regimen transition
2025	Vaccine	BCG-PRIME Phase 2b VE −3.8% (95% CI −37 to 21)	(10)	BCG revaccination redirect
2025	Trial	endTB-Q FQ-resistant 6–9 month favorable signal	(9)	FQ-R alternative
2025	Trial	DRTB-HDT five-country RCT initiated	(13)	First DR-TB HDT trial
2025	Diagnostic	Nanopore direct-from-sputum sequencing operational	(28)	Clinical-timescale DST
2025	Vaccine	MTBVAC Phase 2a infant readout	(11)	Newborn-route validation
2025	Surveillance	CRyPTIC quantitative MIC compendium 151,211 isolates	(30)	Catalogue v3 substrate
2026	Vaccine	M72/AS01E Phase 3 enrollment ~20,000 complete (11 mo ahead)	(82)	2027–2028 readout pending
2026	Policy	WHO Catalogue of Mutations v3 development	(95)	BDQ/PMD/LZD grade update

In the Phase 2b trial involving 1,836 South African adolescents, the participants received either the test vaccine BCG or placebo under the same design/layout as in the Phase 2a trial (10). The trial demonstrated no difference between the two groups’ rates of *M. tuberculosis*; however, there was a significant estimate of −3.8% (95% CI −37 - 21) for the efficacy of the BCG vaccine for the prevention of sustained *M. tuberculosis (*80, 81) infection. These findings also did not align with Phase 2a’s estimate of 45% for sustained *M. tuberculosis* infections, which gave warning enough to discontinue any further evaluations into BCG revaccination as a successful stand-alone strategy for adolescents. Therefore, India, Indonesia, and Pakistan, as part of their respective programs currently under development alongside this research, are researching alternate methods to stimulate protective immune responses. Evaluation of studies on trained immunity found there was insufficient NK cell memory created from KLRG1-+ vaccinated participants to provide protection against *M. tuberculosis* infections. Investigational and regulatory cell-aware recombinant products, including VPM1002 (urease-deficient and listeriolysin expressing), and cyclic-di-AMP over-expressing rBCG, hold promise as whole-cell alternatives and are currently undergoing further investigation as potential Phase 2/3s.

Approximately 20,000 participants were enrolled in the Phase 3 trial of the M72/AS01E vaccine across Indonesia, Kenya, Malawi, South Africa, and Zambia as of February 2026, with recruitment completed eleven months ahead of schedule. The M72/AS01E Phase 2b efficacy estimate of 49.7% supports further evaluation of this subunit vaccine but does not establish a regulatory-cell-mediated mechanism (82, 83).

MTBVAC (live bioengineered Mtb minus deletions of *phoP* and *fadD26*) is undergoing an open-label newborn study (Phase 3) in several countries (South Africa, Madagascar, and Senegal) and an open-label program for adolescents and adults (IMAGINE Phase 2b). In the Phase 2a study by Tameris et al., “Newborns vaccinated with MTBVAC produced greater numbers of CD4 T cells that secreted γ,” than controls (BCG). The IMAGINE program is the primary comparator for the M72/AS01E subunit program (11, 84); the M72/AS01E program is not scheduled to read out prior to 2028. The BNT164a1 and BNT164b1 mRNA-LNP vaccines (BioNTech) began Phase 1 trials in 2025, using combination multi-covalent formulations of ESAT-6, CFP-10, and Ag85B, scheduled to read out in 2026 and 2027, respectively. ID93 + GLA-SE and H56 (IC31 [Phase 2/2b]) are the only therapies in development; H107e/CAF10b (cationic liposome-derived subunit vaccine) is continuing development through Phase 1 (76). The full candidate pipeline (Platform/Antigen(s)/Adjuvant/Indication(s)/Company responsible for candidate) can be found in **Supplementary Table SB-1** (12).

The negative BCG-PRIME result conflicts with earlier estimates of benefit and does not establish a regulatory-cell explanation for vaccine failure (10). Likewise, findings for M72/AS01E and MTBVAC arise from distinct platforms and should not yet be interpreted as evidence of a shared regulatory-immune mechanism. Future readouts may permit comparative analyses across these platforms, but whether regulatory-immune phenotypes predict protection remains unresolved.

## 9 Branch 6: clinical translation and treatment outcomes

### 9.1 WHO 2024–2025 BPaLM scale-up

On the updated consolidated guidelines (WHO 2024) issued for MDR and RR-TB, BPaLM is now designated as the preferred first line of treatment, with BPaL reserved for use in the treatment of fluoroquinolone-resistant MDR and RR-TB, and BDLLfxC for use in the treatment of MDR in pregnant women and for patients with contraindications to BPaLM (**Figure 6**) (85). BPaLM implementation increased from 5,653 patients in 2023 to 34,000 patients in 2024 (6x increase). Activity-based patient care programs exist in 47 of 95 countries (86, 87). The NTEP (National TB Elimination Program) in India initiated BPaLM treatment for 12,000 patients across 23 states in 2024, resulting in a 6-month treatment completion rate of 78%. ATS/CDC/ERS/IDSA (2025) will broaden the recommendation by including BPaLM as a first line of therapy for North America and Europe. Cost effectiveness data from 12 high-burden countries and Moldova indicate that BPaLM will be cost saving in low and middle-income countries, with the key barriers to accessing BPaLM being diagnostic infrastructure, drug-supply logistics, and programmatic capacity versus the cost of the drug (50, 88).

**Figure 6:**
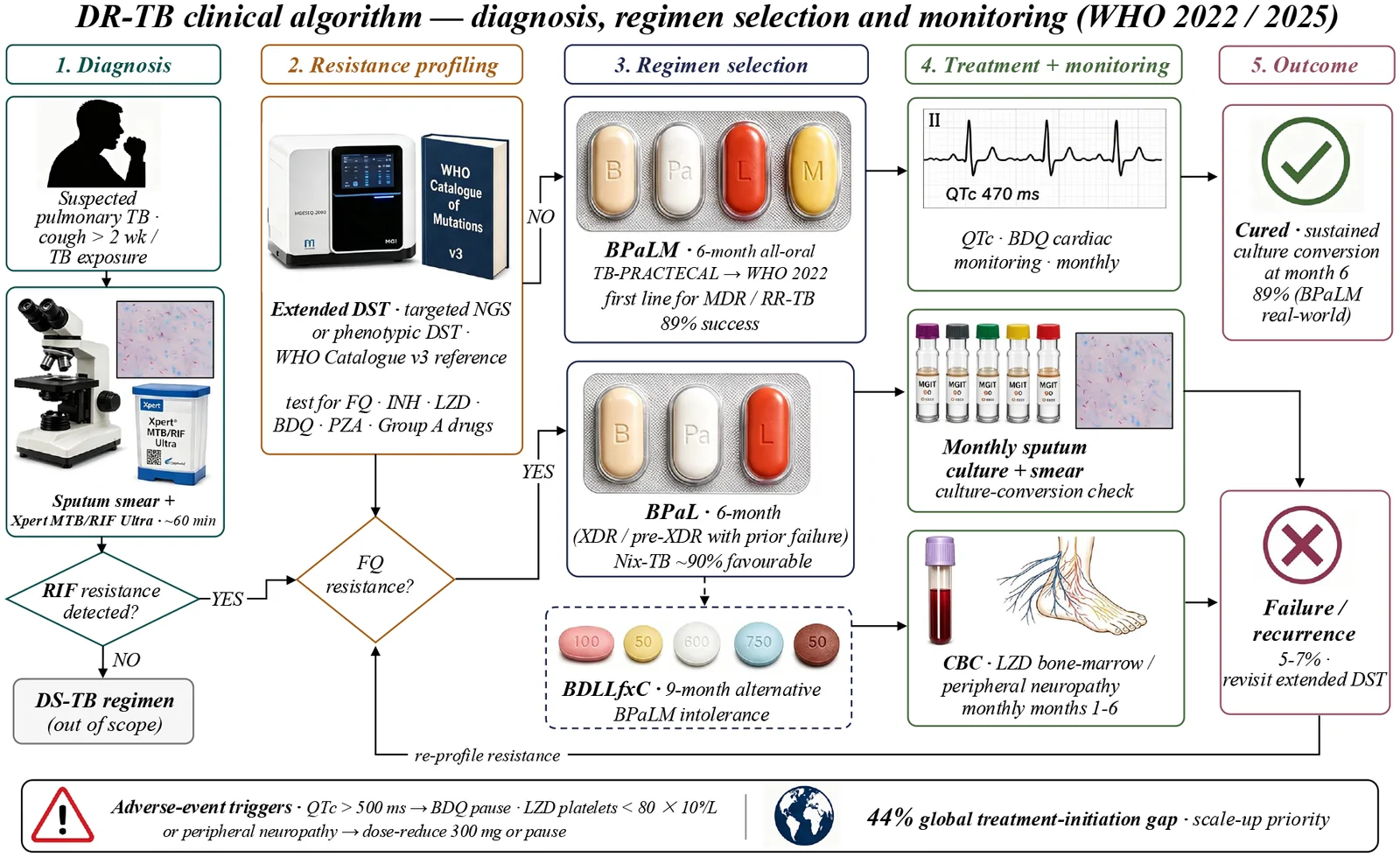
DR-TB clinical algorithm: diagnosis, regimen selection, and monitoring.

### 9.2 Multi-center clinical evidence base

The TB Portals’ data exploration tool integrates clinical, laboratory, radiological, and genomic data from more than 33,000 patients across 40 sites in 16 countries worldwide. 66% of the total number of cases occur as MDR/XDR-TB (89–91). The CRyPTIC consortium consists of 15,211 isolates collected from 23 different countries; of these, 12,289 have phenotype and genotype data available, in addition to quantitative minimum inhibitory concentration (MIC) data for 13 different anti-TB drugs. The CRyPTIC study has isolated 4,004 specimens from India, 2,691 from Peru, 2,155 from South Africa, 1,288 from Vietnam, and 1,121 from China (23, 30). The ReSeqTB dataset expands the database of genotype/phenotype training data that WHO Catalogue v2 will be built upon. EndTB provides 754 patients from seven countries taking five all-oral combinations, and endTB-Q provides an additional 324 patients from their respective countries (8). TB-PRACTECAL dataset gives evidence of BPaLM with 552 patients across four countries. STREAM Stage 1 & Stage 2 confirm that the nine-month regimen is non-inferior to the historical 20-month regimen, with 78.8% success across eight countries. IeDEA and the TB Sentinel Research Network provide longitudinal data on HIV (co-infection) with TB from a multi-country study across five southern African countries (see **Table 5**) (24, 92).

**Table 5:** Multi-center tuberculosis databases and cohorts informing the evidence base of this review.

Resource	Year span	Size	Geographic coverage	Data modality	Availability
TB Portals (NIAID)	2008–2025	331,000+ patients (16 countries, 40 sites)	Asia, E. Europe, Africa, Americas	WGS + phenotypic DST + radiology + outcomes	Registered user
CRyPTIC Consortium	2017–2024	151,211 isolates (23 countries; 121,289 paired)	Africa, Asia, Europe, Americas	WGS + quantitative MIC (13 drugs)	Open
ReSeqTB (CPTR)	2014–2025	221,000+ isolates (20+ countries)	Global	WGS + phenotypic DST	Open (registered)
WHO Global TB Programme	1995–2025	215 national programs; ~10.8M cases/yr	All WHO regions	Surveillance; outcomes	Open
TB-PRACTECAL (MSF)	2017–2023	552 patients	Belarus, South Africa, Uzbekistan, Pakistan	RCT (BPaLM vs SOC)	Open
endTB/endTB-Q	2017–2025	754 + 324 patients	7 countries	RCT (five all-oral arms)	Open
STREAM 1/2	2012–2022	424 + 588 patients	8 countries	RCT (nine-mo non-inferiority)	Open
IeDEA TB-SRN	2022–2025	~11,800	5 Southern African countries	HIV/TB longitudinal cohort	Registered
Afro-TB dataset	2010–2025	131,753 strains	Africa	WGS + DST	Open
DRTB-HDT trial	2024–2026	~330 patients (planned)	Romania, Moldova, Georgia, Mozambique, South Africa	RCT (HDT adjunct)	Open
BCG-PRIME (NEJM 2025)	2020–2024	11,836 adolescents	South Africa	RCT (BCG revacc)	Open
M72/AS01E Phase 3	2024–2028	~20,000 adolescents/adults	Indonesia, Kenya, Malawi, South Africa, Zambia	RCT (Phase 3)	Restricted

### 9.3 Minimum clinically important differences and subgroup heterogeneity

Anchored by the non-inferiority margins defined by regulatory agencies (as well as pooled trial data), the MCID equal to endpoints DR-TB is evident. For example, with regard to the success of treatment in subjects from the study on linezolid, a statistically significant difference of 12 percentage points (from 89 percent for all oral to 52 percent for injectable) exists between the time point of treatment success and failure; this difference exceeds the approved non-inferiority margin (30, 93). Other studies also document such benefits: culture conversion was 30 days earlier and mortality was reduced from 16.4% to 7.5%, a 54% relative reduction (45). Concordance between the diagnosis of both quantitative and qualitative criteria for diagnosing MIC [first-line drugs based on bedaquiline and linezolid that have been lengthened due to unresolved mutations] are both demonstrated with thresholds of 96 to 98 percent (quantitative) and 89 to 93 percent (qualitative). At least eight different aspects regarding the heterogeneity of treatment response to zBM seen at the time of CD4 and/or recognizing (73% of 30,000 new pediatric CD4 cases/year— the serial access barriers to child-friendly formulations) have been identified across strata based on ARV resistance-85 percent MDR, 75 percent XDR (XDR), and 65 percent XDR (by sedation), sex (by location), diabetes history [controlled OR of unsuccessful outcome = 1.56], disease site and degree of impairment to lung function after TB [*>* after 30 percent and *><*60 percent of patients successfully treated]. Treatment history prior to CD4 initiation due to changes in the extent of changes in productive capacity after initiation of the final approval criteria, unless lost due to the number of changing diagnoses and respective treatment protocols, were allowed to be published.

### 9.4 Equity, the 44% treatment-initiation gap, and the bedaquiline-resistance surveillance imperative

In 2023, only 176,000 (44%) of the 400,000 predicted cases of DR-TB initiated treatment—primarily due to limited diagnostic capacity in sub-Saharan Africa, funding shortfalls in post-Soviet states across Eastern Europe and Central Asia, and disruptions among refugee populations in conflict zones. The bulk of the global burden of disease (56%) is represented by five nations: India, Russian Federation, Indonesia, China, and Philippines. However, there is much variability in terms of access to diagnosis and therapies within these five countries. At this time, the use of dispersible pediatric tablets for BPaLM has yet to be approved. Pregnant individuals may not receive pretomanid; therefore, it is much more challenging to administer BDLLfxC as a programmatic solution compared with DR-TB. Studies such as TB-CHAMP and VQUIN have provided evidence that levofloxacin will prevent transmission of MDR from persons exposed to MDR, thereby providing another avenue to impede transmission and eliminate the diagnostic gap through interrupting transmission (1, 92, 94).

Surveillance for bedaquiline resistance is currently the highest-priority operational focus of BPaLM durability in terms of operational priority. Whole-genome sequencing determined that DST is 100% concordant with first-line drugs and 89-93% concordant with bedaquiline and linezolid; therefore, the nanopore direct-from-sputum methods and one-pot CRISPR-Cas12 assays provide the means for real-time adjustment of the treatment regimen in low-resource settings (28). Branch 4 suggests candidate immune phenotypes for future precision-adjunctive studies, including Treg/MDSC ratios and exhausted-CD8 profiles (Section 11). Their clinical utility and the feasibility of a multicenter trial remain untested.

## 10 Discussion: cross-cutting themes

### 10.1 Resistance–immunity bidirectional reinforcement

Seven convergent but predominantly indirect lines of evidence motivate a bidirectional-reinforcement hypothesis; however, their heterogeneity across models, populations, and endpoints precludes causal inference (42). Discordant vaccine findings, the lack of randomized validation of immune-directed adjuncts, and plausible alternative explanations for residual treatment failure mean that the proposed loop and its intervention implications are hypothesis-generating rather than established or dominant mechanisms (6, 10, 13).

### 10.2 Precision adjunctive therapy by host immune phenotype

The proposed model could stratify patients by regulatory-immune phenotype, with candidate interventions matched to Treg-high, MDSC-high, or exhausted-CD8 profiles. These matches remain investigational and require prospective validation before informing treatment selection. Basket or umbrella trials could test these hypotheses across resistance strata, and the DRTB-HDT network may offer infrastructure for such evaluation (13).

### 10.3 Geographic diversity, computational tools, and equity as a design criterion

Data for the proposed project have been gathered in over 22 countries across the six WHO regions; this includes groups of participants from South Africa, Nigeria, and Mozambique as the primary sources of evidence in Africa, along with groups from India, Vietnam, China, Pakistan, and Indonesia that provide examples of evidence from Asia. In Eastern Europe, groups of participants from Belarus, Moldova, Ukraine, Romania, and Georgia provided data; while in the Americas, groups from Peru served as a source of evidence. There are currently two direct gaps in this data: Brazil and Mexico. These gaps are expected to be addressed through the formation of the Pan American Health Organization; Regional Research Network. The computational tools created for this project include TB-Profiler, integrated with WHO Catalogue v2, Mykrobe to handle nanopore outputs, gnomonicus to identify mixed-strain samples, TBtypeR for quantitative MIC machine learning extensions, a federated learning architecture that spans TB Portals, CRyPTIC, ReSeqTB, and IeDEA, as well as a target validation pipeline that is supported by AlphaFold. All of these computational tools will provide clinical decision support within resource-limited settings at the time of operational deployment of the molecular surveillance process (95). Therefore, the gap of 44% in the global initiation of treatment cannot be attributed to an external constraint; they rather reflect an internal design specification: sites to evaluate trials, choices of adjuvants, and combinations of HDTs used in pivotal studies must be capable of deploying in resource-limited settings from the outset and not exclusively at high-resource reference sites (96).

## 11 Open problems and future directions

Based on named milestones from 2026 to 2030 we can identify nine open problems related to each milestone (see **Supplementary Table SC-1**). These priorities reflect recurrent evidence gaps, unresolved controversies, feasibility, and the authors’ expert judgment.

As of October 2023, an estimated 20,000 adolescents and adults will continue to receive long-term sustainable protection from IGRA across five countries with a high burden of disease. Therefore, there are no clinical data available or anticipated to support the submission to regulatory authorities for the purpose of AS01E with a regulatory milestone strategy (82).

OP-2: MTBVAC IMAGINE Phase 2b readout (2028). The IMAGINE trial is expected to enroll approximately 4,300 adolescents and adults in South Africa, Kenya, and Tanzania to evaluate whether MTBVAC prevents pulmonary TB disease. This adult/adolescent program complements the separate Phase 3 newborn program in South Africa, Madagascar, and Senegal (11, 84).

OP-3: BNT164a1/b1 mRNA TB vaccine Phase 1 (2026–2027). This trial may inform regulatory-cell-aware design, but it cannot validate that framework without direct immune-mechanistic endpoints.

The proposed trial will compare metformin or dovramilast plus standard care with standard care alone in approximately 330 patients with RR-TB. Its FEV1 and culture-conversion endpoints may test selected framework predictions but cannot validate the bidirectional-reinforcement model by themselves (12, 13).

No randomized trial has evaluated Treg- or MDSC-directed adjunctive therapy for DR-TB. A proposed basket design could stratify MDR, pre-XDR, and XDR participants by Treg/MDSC signatures, but its feasibility and sample-size assumptions remain to be established. The protocol for the study is expected to be published in 2027 and the primary outcome data will be available from 2029-2030.

OP-6. WHO Catalogue v3 (WHO Catalogue v3) has initiated bedaquiline (bedaquiline), pretomanid (pretomanid), and linezolid (linezolid) implementation and integration into the clinic between 2027-2028. The purpose of OP-6. WHO Catalogue v3 (WHO Catalogue v3) is to provide a resolution for the unresolved mutations at *atpE* (*atpE*), *Rv0678* (*Rv0678*), *pepQ* (*pepQ*), *ddn* (*ddn*), and *fbiA-D* (*fbiA-D*), as well as *rrl* (*rrl*) and *rplC* (*rplC*); all mutations will require AI-based catalog integration and federated WGS for continual monitoring of the patients’ progress. As such, it is anticipated that patients who have experienced a relapse, have sustained lung injury post-TB (TB), and/or have developed relapse-acquired-resistance from TB Portals (TB Portals) and endTB (endTB) over the longitudinal study period (2027-2029) will have high rates of recurrence.

OP-8: AI for resistance prediction in clinical workflows. Population benchmarking and AlphaFold (AlphaFold) will continue to be integrated with EHRs from underserved regions and will not exacerbate the access-to-diagnosis barriers (97). Finally, there remains a need for creation of a research and implementation program agenda across diagnostic systems in Sub-Saharan Africa, the programmatic capacities in Eastern Europe, access to clinical services in Southeast Asia, and anti-stigma frameworks creating trajectories from End TB 2030 (End TB 2030) (95).

## 12 Limitations

The five limitations in this synthesis were deliberately designed and characterized by the sufficiency-scope-rationale-position-forward plan framework rather than deficiencies in the design. First, this work is a narrative–systematic hybrid rather than a meta-analysis. Between-study heterogeneity precludes reliable pooled estimates, although the 350 eligible studies support qualitative synthesis across branches. Second, while branch-specific Cochrane reviews are being planned for use in 2027-2028, the literature review associated with this synthesis will close mid-2026; completion of the outstanding Phase 3 readouts (M72/AS01E, MTBVAC and IMAGINE) will be after the closure date, with the date of the last preprint supporting the forward edge of the synthesis being the latest addition to the synthesis; the author plans to update the evidence from this synthesis in 2027-2028. Third, the synthesis includes primary literature published in indexed English, Russian, Mandarin, and Indonesian publications; the primary literature published by these countries regarding the global burden of MDR-TB for these particular drug regimens is currently only partially represented by the respective multinational consortia (CRyPTIC, TB Portals, and WHO catalogs); therefore, these countries are lacking in evidence. The author intends to create a multi-lingual evidence clustering framework to be used in the updated evidence synthesis planned for 2027-2028, which will be developed by 2025. Fourth, real-world evidence communicating the prevalence of recurrence and post-trauma lung damage for BPaLM for years 2 and 5 from 18 implementation cohorts is still being collated; therefore, interim analyses will be performed at the 15-year trend mark for efficacy and the 15-year long-term extension of TB-PRACTECAL until dedicated reviews of the corresponding outcomes focusing on the years 2027–2028 have been completed. Fifth, the synthesis focuses primarily on the 22 anchor countries; however, the lowest weight of evidence is based on countries with high-burden cases and minimal resources, namely Brazil and Mexico. Given the operational urgency of developing countries with high-burden cases, weighting the evidence according to countries’ burden would seem most appropriate; therefore, the author also plans to create a Latin America synthesis in 2027 to reduce the gap between the regions of the world in the availability of primary evidence.

## 13 Conclusion

DR-TB involves both microbial resistance and host immune responses, but current evidence does not establish a causal feedback mechanism between them. The bidirectional-reinforcement framework should therefore be viewed as an integrative hypothesis for future testing rather than a definitive account of treatment failure. The following five recommendations arise from this. The first is that routine whole-genome sequencing, and WHO Catalogue v2 integration through TB-Profiler, Mykrobe, and AI-enhanced decision support, become the clinical standard for DR-TB diagnosis. The second is that BPaLM patient population constitutes the six-month all-oral standard of care, provided that bedaquiline resistance is monitored and an operational response developed that utilizes combinations of novel diarylquinolines, mechanism-unrelated regimens, and adjunctive HDT. The third is that, for HDT to be integrated into standard of care, DRTB-HDT primary readout results and follow-on basket trials will provide the foundational evidence for the first DR-TB-specific Treg/MDSC modulator combination. Fourth, regulatory-immune criteria should be evaluated alongside established clinical evidence when selecting vaccine and adjuvant candidates. Finally, the scale-up to equitable elimination of the 44% treatment initiation gap is by design and not an afterthought. A complete empirical infrastructure (TB Portals, CRyPTIC, ReSeqTB, endTB, TB-PRACTECAL, STREAM, DRTB-HDT) exists for testing, refinement, and deployment of the bi-directional framework against the End TB 2030 milestones trajectory.

## 14 Theoretical framework: formal articulation of the bidirectional reinforcement model

This formalization encodes testable assumptions rather than empirically established causal relations.

Let ℛ(t) denote the Mtb reservoir size, S(t) the intensity of the host regulatory-immune program (composite of Treg, MDSC, and exhausted-T-cell density in the granuloma compartment), D(t) the cumulative drug-resistancemutation acquisition rate, and V(t) the magnitude of protective vaccine effector immunity. The DR-TB failure system is *bidirectionally reinforcing* if and only if (i) ∂S/∂ℛ>0 (a larger reservoir sustains stronger regulatory-immune suppression through chronic antigen load); (ii) ∂ℛ/∂S>0 (stronger regulatory-immune suppression enables a larger persister reservoir by dampening effector clearance); (iii) ∂lV/∂S<0 (regulatory-immune suppression dampens vaccine effector immunity); and (iv) ∂D/∂ℛ>0 (a larger reservoir under selective pressure accumulates more resistance mutations per unit time). Empirical anchors include (i) granuloma single-cell and spatial transcriptomic atlases; (ii) the checkpoint-driven CD4 dysfunction–recrudescence cohort (42); (iii) the BCG-PRIME negative readout and M72/AS01E Phase 2b combined; and (iv) WHO Catalogue v2 and lineage-stratified resistance trajectories. Within this conceptual model, the potential value of multi-axis intervention is a proposition for empirical testing rather than a clinical requirement.
