# Supplementary material for: Novel strategies against drug-resistant tuberculosis: a comprehensive review from molecular mechanisms to clinical translation
Source: Front Immunol. 2026 Oct 21;17:1912284. doi: 10.3389/fimmu.2026.1912284 (PMC13732310; doi:10.3389/fimmu.2026.1912284)
Supplement: Supplementary file 1 [file Supplementaryfile1.pdf]

Supplementary Material

Novel Strategies Against Drug-Resistant Tuberculosis:
A Comprehensive Review from Molecular Mechanisms to Clinical Translation

Supplementary Material

The following supplementary tables (SA-1–SC-1) serve as confirmation of the information provided in the main body: SA-1 is an example of the WHO Catalogue v2 mutation records that are included in this catalog (i.e., drug, gene, position, frequency, severity), as well as a catalog of all the mutation records which can be accessed at https://www.who.int/publications/i/item/9789240082410; SA-2 shows the number of records in each stage of the PRISMA flow from 858 identified records to 350 included studies; SB-1 provides a table listing the 22 TB vaccine candidates organized by platform, antigen, adjuvant, phase, and sponsor; SB-2 gives the agents and mechanisms of action for the host-directed therapy candidates, as well as preclinical data showing efficacy, current clinical phases, and whether or not any potential interactions exist with the agent; SB-3 has a list of immunomodulatory agents organized by their cellular targets, mechanisms of action, method of developing them by platform, levels of evidence, and current stage of development; and SC-1 is a timeline of the OP-1 through OP-9 milestones as discussed within the text.

Table 1: Supplementary Table SA-1: representative WHO Catalogue v2 (2023) mutation entries (illustrative subset).

Drug	Gene	Position / mutation	Frequency in CRyPTIC + ReSeqTB	WHO grade (v2)	Notes
Rifampicin	*rpoB*	S450L	35–45% of RR isolates	1 (Assoc. w/ R)	Most common RR allele globally
Rifampicin	*rpoB*	D435V	8–12% of RR	1 (Assoc. w/ R)	Common in Beijing lineage
Rifampicin compensatory	*rpoA*/*rpoC*	Various	∼20% of RR	N/A (fitness)	Restores transmissibility
Isoniazid	*katG*	S315T	65–80% of INH-R	1 (Assoc. w/ R)	Most common INH-R allele
Pyrazinamide	*pncA*	Various LoF	>80% PZA-R	1 (Assoc. w/ R)	Wide allelic spectrum
Fluoroquinolone	*gyrA*	D94G/N/A/Y /H	70–85% FQ-R	1 (Assoc. w/ R)	Codon 94 dominant
Bedaquiline	*Rv0678*	Promoter / coding	Cape Town 22–47% acquired	2 (interim)	MmpL5 efflux; CFZ cross-R
Bedaquiline	*atpE*	A63P, I66M, E61D	∼5% global; Russian dominance	2 (interim)	High-MIC; binding pocket
Bedaquiline	*pepQ*	A55V, frameshift	Cape Town 13%	3 (uncertain)	Co-occurs with *Rv0678*
Pretomanid / Delamanid	*ddn*	W88R, R72H	30–50% of PMD-R	2 (interim)	F420 activation loss
Linezolid	*rrl* (23S rRNA)	G2576T	∼70% acquired LZD-R	1 (Assoc. w/ R)	High-MIC
Linezolid	*rplC*	C154R	∼20% LZD-R	2 (interim)	Low-level LZD-R
Aminoglycoside	*rrs*	A1401G	70–90% AK/KM-R	1 (Assoc. w/ R)	Cross-resistance AK/KM
Telacebec (Q203)	*qcrB*	T313I, A317V	Preclinical	Pipeline	Not in catalogue v2
BTZ-043 / DprE1	*dprE1*	C387S etc.	Preclinical	Pipeline	Not in catalogue v2

Table 2: Supplementary Table SA-2: PRISMA flow numerics.

Stage	n	Notes / exclusion reasons
Records identified (databases)	581	PubMed/MEDLINE 412, Embase 38, Web of Science 95, Scopus 22, Cochrane CENTRAL 14
Records identified (other sources)	277	Preprints 14; registries 50; citation chase 142; targeted searches 69; advocacy 2
Total records identified	858	Sum of databases + other sources
Records after deduplication	612	246 duplicates removed
Records excluded at screening	198	Outside 6-branch scope 87; non-Mtb 18; non-peer-reviewed 14; predatory/retracted 6; other
Full-text articles assessed	414	Per protocol
Full-text excluded	64	DOI unverifiable 24; insufficient DR-TB outcome 18; animal-only 9; redundant 13
Studies included in qualitative synthesis	350	Cross-branch synthesis substrate
Currency (2024–2026)	207 (59.1%)	Exceeds 40% threshold for fast-moving field
Distinct countries cited	22	All 6 WHO regions anchored

Table 3: Supplementary Table SB-1: TB vaccine pipeline (selected candidates) by platform, antigen, adjuvant and phase.

Vaccine	Platform	Antigens	Adjuvant / vector	Phase	Indication	Sponsor
BCG (Pasteur)	Live-attenuated	Whole organism	Live	Approved 1921	Newborn primary	WHO EPI
BCG-PRIME revacc	BCG revacc	Whole organism	Live	Phase 2b (NEGATIVE)	Adolescent IGRA−	Gates MRI / SATVI
VPM1002	Recombinant BCG	Whole + listeriolysin	Live	Phase 2/3	Newborn alt; PLWH	Serum Institute India
M72/AS01E	Subunit	Mtb32A + Mtb39A	AS01E (saponin + MPL liposomal)	Phase 3 complete	IGRA+ adolescent/adult	GSK / Gates MRI / Wellcome
H56:IC31	Subunit	Ag85B + ESAT-6 + Rv2660c	IC31 (TLR9)	Phase 2b	Reinfection prevention	SSI / Aeras
ID93 + GLA-SE	Subunit	Rv2608, Rv3619, Rv3620, Rv1813	GLA-SE (TLR4)	Phase 2	BCG-vaccinated adult	IDRI / AAHI
MTBVAC	Live-attenuated Mtb	Whole organism (*phoP*/*fadD26* del)	Live	Phase 3 newborn + Ph 2b IMAGINE	Newborn primary; adolescent BCG alt	Biofabri / IAVI / Gates
BNT164a1/b1	mRNA-LNP	Multi-antigen (ESAT-6, CFP-10, Ag85B)	LNP intrinsic	Phase 1	Adult	BioNTech
DAR-901	Inactivated whole cell	*M. obuense*	Inactivated	Phase 3 anticipated	Adolescent / adult	Dartmouth + partners
TQL1055 saponin	Subunit + saponin adjuvant	Mtb fusion antigen	TQL1055 saponin	Preclinical	Adult	Adjuvance Tech
Mucosal Ad5-Rv2299c	Adenovirus 5 vector	Rv2299c	Vector intrinsic	Phase 1	Airway-targeted	Academic consortium
PVR Fc-engineered Ab	Passive immunotherapy	Engineered Mtb-binding IgG	Fc engineering	Preclinical	Adjunct	Harvard / Ragon
Mn^{2+} + H56	Subunit + cGAS-STING	Mtb antigens + Mn^{2+}	Mn-cGAS-STING	Preclinical	Adult	Academic consortium
Cabrera trans-sialidase TB adaptation	Subunit + MDSC modulation	Trans-sialidase analogue	Treg/MDSC-aware	Preclinical	Adolescent / adult	Cabrera laboratory

Table 4: Supplementary Table SB-2: host-directed therapy candidates for drug-resistant tuberculosis.

Agent	Target / mechanism	Mtb-relevant preclinical signal	Clinical phase	Potential interaction	Branch link
Metformin	AMPK; autophagy; macrophage polarisation	Murine reduction; cohort lower mortality	DRTB-HDT RCT (5-country)	BPaLM backbone	3 + 4
Dovramilast	PDE4; cAMP; macrophage repolarisation	DRTB-HDT preclinical	DRTB-HDT RCT (5-country)	BPaLM backbone	3 + 4
Vitamin D	Cathelicidin; autophagy	SCC acceleration in deficient pts	Approved supplement	Any regimen + nutritional gap	3
Rosuvastatin	HMG-CoA reductase; macrophage lipid metabolism	STAT-TB 2023 RR-TB adjunct trial	Phase 2	Any DR-TB regimen	3
Anti-PD-1 (stratified)	Checkpoint blockade	Murine partial benefit; reactivation paradox	Preclinical / case reports	Stratified by phenotype	3 + 4
Anti-TIGIT	TIGIT blockade	Murine bacterial control	Preclinical	BPaLM + biomarker-stratified	3 + 4
OX40 agonist	CD4 costimulation	Murine HDT signal	Preclinical	BPaLM + immune phenotype	3 + 4
Fc-engineered antibody	Neutrophil-mediated Mtb control	Preclinical Mtb control	Preclinical	BPaLM + Fc engagement	3 + 5
ATRA	MDSC differentiation	Preclinical reduction in granuloma MDSC	Preclinical / Phase 1 design	BPaLM + regulatory-cell modulator	3 + 4
Low-dose 5-fluorouracil	MDSC depletion (sub-cytotoxic)	Preclinical depletion	Preclinical / Phase 1 design	BPaLM + regulatory-cell modulator	3 + 4
Anti-CD25 (basiliximab / daclizumab)	Treg depletion	Preclinical depletion in murine models	Approved transplantation; preclinical TB	BPaLM + transient Treg depletion	3 + 4
Tadalafil	PDE5; MDSC modulation in oncology	Preclinical promising	Preclinical	BPaLM + Treg/MDSC modulator	3 + 4

Table 5: Supplementary Table SB-3: regulatory immune modulators with potential drug-resistant tuberculosis application.

Modulator	Target cell	Mechanism / pathway	Platform	Preclinical / clinical signal	Development status
Anti-CD25 (basiliximab)	Treg	IL-2Rα depletion of high-Foxp3 Treg	Monoclonal antibody	Transient depletion preclinical; oncology repositioning	Approved transplant; TB preclinical
All-trans retinoic acid (ATRA)	MDSC	Granulocyte differentiation	Small molecule	Preclinical MDSC reduction in murine granuloma	Preclinical / Phase 1 design
Low-dose 5-FU	MDSC	Sub-cytotoxic depletion	Generic chemo	Preclinical depletion	Preclinical / Phase 1 design
Tadalafil	MDSC	PDE5 / cGMP–cAMP signalling	Approved oral	Oncology Phase 2 PDE5–MDSC; preclinical TB	TB preclinical; oncology approved
Anti-TIGIT	Exhausted CD8 + Treg	Co-inhibitory checkpoint blockade	Monoclonal antibody	Preclinical bacterial control	Preclinical
Anti-CTLA-4	Treg	Co-inhibitory checkpoint blockade	Monoclonal antibody	Lower TB reactivation than anti-PD-1	Approved oncology; preclinical TB
Anti-PD-1 (stratified)	Exhausted CD8 + Treg	Co-inhibitory checkpoint blockade	Monoclonal antibody	Murine partial benefit; clinical reactivation paradox	Approved oncology; TB case reports
Manganese (Mn^{2+}) adjuvant	Innate (cGAS-STING)	cGAS sensitisation; STING activation	Vaccine adjuvant	Preclinical + early-clinical cancer-vaccine	Preclinical TB / cancer Phase 1
Saponin (TQL1055, QS21)	DC + Treg modulation	TLR4 + NLRP3 + saponin chemistry	Vaccine adjuvant	Preclinical Mtb protection	Preclinical to Phase 1
TLR9 ODN (IC31)	DC + Th1 polarisation	TLR9 engagement	Vaccine adjuvant	Phase 2b H56:IC31 reduced reinfection	Phase 2b clinical
GLA-SE	DC + Th1 polarisation	TLR4 engagement; effector polarisation	Vaccine adjuvant	Phase 2 ID93 immunogenicity	Phase 2 clinical
LNP (intrinsic adjuvant)	Innate (TLR, RIG-I)	Innate sensing + Th1 polarisation	mRNA delivery	Phase 1 BNT164a1/b1	Phase 1 clinical
OX40 agonist	Recently activated CD4	Co-stimulation; effector amplification	Monoclonal antibody / agonist	Preclinical murine HDT signal	Preclinical

Table 6: Supplementary Table SC-1: Open Problem milestone timeline (OP-1 through OP-9).

OP	Topic	Anticipated readout	Anchor study / framework
OP-1	M72/AS01E Phase 3 efficacy	2027–2028	NCT06062238
OP-2	MTBVAC IMAGINE Phase 2b	2028	NCT04975178 + IMAGINE
OP-3	BNT164a1/b1 mRNA Phase 1	2026–2027	BioNTech preprint 2025
OP-4	DRTB-HDT 5-country primary endpoints	2025–2027	NCT04504435
OP-5	First DR-TB-specific Treg/MDSC modulator combination trial	2029–2030	Basket design; n=150–200/arm; ≥4 WHO regions
OP-6	WHO Catalogue v3 BDQ/PMD/LZD clinical-grade integration	2027–2028	WHO Catalogue v3 + AI interface
OP-7	BPaLM 2- and 5-year real-world outcomes	2027–2029	TB Portals + endTB long-term extension
OP-8	AI for resistance prediction in clinical workflows	2026–2028	TB-Bench benchmark; federated learning
OP-9	Equity-aware deployment science	End TB 2030 trajectory	Lancet Global Health implementation framework
